# The role of humanitarian missions in surgical training for maxillofacial surgery residents: SOS Face Marrakesh experience

**DOI:** 10.11604/pamj.2021.40.160.31964

**Published:** 2021-11-16

**Authors:** Zakaria Aziz, Salma Aboulouidad, Abdelghafour Jaifi, Mohamed El Bouihi, Nadia Mansouri Hattab, Hanane Rais

**Affiliations:** 1 Maxillofacial Surgery Department, University Hospital Center Mohammed VI, Marrakech, Morocco,; 2 Anatomic Pathology Department, University Hospital Center Mohammed VI, Marrakech, Morocco

**Keywords:** Humanitarian work, surgical training, maxillofacial resident, educational portfolio

## Abstract

Resident´s participation in humanitarian work has been controversial, while it´s recognized by some authors to bring significant value to the resident´s education. Herein, we aim to provide an evidence of the role of humanitarian missions in the surgical training as part of residency program, through report of a 10 years experience of SOS FACE Marrakesh, a non-benefit association within maxillofacial surgery department of Marrakesh. Its operating mode is to organize humanitarian missions coupled to targeted surgical training program, which is framed by educational objectives using a pedagogic portfolio. As a result, 60.6% of the residents felt an improvement in surgical skills, and the evaluation of residents before and after the targeted training showed an increase of 57% in average clinical knowledge, especially the diagnosis part. In conclusion, humanitarian work helps to improve surgical skills in addition to enhancement of human values and we suggest incorporating volunteerism in residency programs.

## Introduction

Marrakesh, as a city in a developing country, requires organization of humanitarian missions to provide health care for disfigured patients who are mostly poor, in need and living in underserved areas. Those campaigns are in the first place for the benefice of the patient. In addition, the maxillofacial surgery department of Marrakesh, using an educational portfolio as a tool for acquiring surgical skills, has always focused its humanitarian actions on an educational dimension. Some authors have recognized that the humanitarian setting allows maximal exposure and learning and can play a significant role in the resident´s education [1]. Herein we aim to highlight the role of humanitarian missions in the surgical training as part of residency program, through report of a 10 years experience of SOS FACE Marrakesh, an association within maxillofacial surgery department of Marrakesh.

## Methods

We conducted a retrospective study reporting the experience of SOS FACE Marrakesh, a non-profit charity association founded in 2009, and based within Ibn Tofail hospital in collaboration with maxillofacial surgery department of Marrakesh. Its fields of action are maxillofacial, oral and plastic surgery.

We included all the campaigns that aimed to treat cleft lip and palate patients, from 2009 to 2019 in Marrakesh. Patients were received at the maxillofacial surgery department for the diagnosis and the preoperative screening in order to establish a surgical schedule, and then the follow-up is carried out on a regular basis in the diagnosis centre of Marrakesh. For the trainees, in particular the residents, the theoretical courses were held at Medical faculty of Marrakesh, while the surgical training took place at the operating rooms of Ibn Tofail hospital using local expertise and material resources.

All the missions along with the educational program were under the supervision of 3 senior surgeons and every action carried out complied with the following protocol: implementation of the concept of targeted surgical training which involves the choice of a thematic adapted to the training schedule of residents as well as the target audience ranging from medical students to certified practitioners; establishment of an educational framework that responds to variable and gradual educational objectives. The latter guide the triage of patients at the consultation, the support of theoretical courses and finally the type of surgical training; based on the year of residency program, the trainees are divided into small groups with a rotation schedule over workshops and 4 operating rooms for the live surgery and assessment of the educational outcome of both the trainee and the trainer at the end of each mission. Participants were surveyed using both Likert-scale and dichotomous questions; an example of it is presented in Annex 1.

## Results

During the study period, a total of 16 humanitarian missions coupled with targeted surgical training occurred for the benefit of 330 patients, mainly coming from rural areas (63%), with nearly 1000 interventions. Each action was run over a one-week period. In total, 40 surgery residents benefited from theoretical and practical training around the management of cleft lip and palate, with an average participation in 4 campaigns per resident. Among them, 13 are currently qualified and autonomous surgeons.

**Figure 1:**
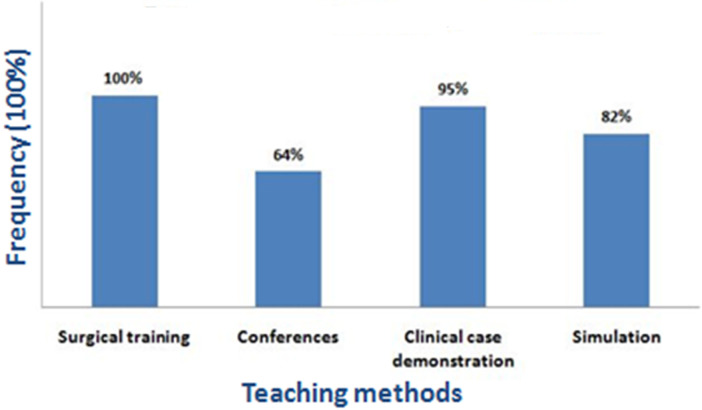
frequency of use of teaching methods during the humanitarian missions

Regarding teaching methods, an educational portfolio was almost regularly applied including: conferences and video conferences with international speakers, clinical case demonstration, simulation and then surgical training where the participants took part of live surgeries (Figure 1). The survey response rate was 65%, which of 92% expressed their desire to come back for another missions and 60.6% felt an improvement in surgical skills. Regarding the teaching methods, the satisfaction degree was mostly excellent (Table 1). The evaluation of residents before and after the targeted training showed a notable increase in average clinical knowledge scores, particularly the diagnosis and surgical indications component (Table 2).

**Table 1:** satisfaction degree of the trainees regarding different teaching methods

Teaching Method	Excellent	Very good	Good	Acceptable	Bad
Clinical case demonstration	75%	16%	6%	1.5%	1.5%
Simulation	83%	10%	3%	2%	2%
Conferences	72%	12%	10%	3.5%	2.5%
Surgical training	86%	13%	0.5%	0.5%	0%

**Table 2:** evaluation of residents before and after the training program following a humanitarian mission

Clinical Knowledge	Before Training	After Training
Fundamental basics	4.35/5	4.87/5
Diagnosis	2/5	4.75/5
Surgical indications	4.25/10	8.37/10

## Discussion

The operating mode of SOS FACE Marrakesh is first individualized by its continual aspect over time, unlike many humanitarian missions which are structured as a “one-off” short-term visit without continuous follow-up, leaving the management of post-operative care and complications to the local teams [1, 2]. This aspect of our missions allows the young surgeon to evolve in his daily practice and to evaluate his learning curve, an example of that is the management of cleft lip and palate sequelae, where at the diagnosis centre, the resident can follow-up all the cases, understands the healing process and discusses management of the complications and sequelae.

Moreover, if the other non-governmental organizations bring with them their own medical and surgical staff, SOS FACE organizes “intra-muros” humanitarian missions calling on local skills and resources, particularly residents.

Resident participation in humanitarian service has been highly controversial and criticized by some as having the potential for unsupervised clinical practice by underqualified trainees [3], while others are concerned about resident´s absence from their training program [4]. To overcome these issues, our missions are part of a university setting following a surgical training program framed by an educational portfolio articulated around a main thematic in each edition. The resident work is always supervised by 3 PhD seniors in maxillofacial and plastic surgery qualified with a pedagogy certificate. The program constantly features:

***Theoretical learning:*** course and lectures given by national and international speakers which allows an exchange of experiences and improves the resident´s communication skills.

***Practical workshops:*** where learning is interactive through audiovisual supports and simulation. During this step, the resident can assimilate the operating technique due to iterative slow motion demonstration by the senior.

***Live surgery:*** every session begins with a briefing, and then the trainee takes the role of an observer and operating assistant, before taking a part of the gesture.

The best evidence of the effectiveness of this humanitarian/pedagogical duality of our missions is 13 residents becoming currently autonomous cleft lip and palate surgeons. The coupling of humanitarian work to targeted surgical training program finds a major interest in orphan pathologies since it ensures the recruitment of patients and facilitates accessibility to the operating rooms, thus filling the lacks in medical knowledge and surgical training. This opinion is shared by many authors [5, 6]. In our research, the evaluation of the trainees before and after the mission showed an increase of 57% in average clinical knowledge especially the diagnosis part.

In addition, the campaigns provide an intensive instruction as it allows the resident to see such a numerous repetitive identical interventions in a short period of time, thus resulting in a better assimilation of the techniques and faster acquisition of surgical skills. This was stated in the Campbell *et al*. work, as 100% of the participants reported that the medical mission was valuable for intensive teaching about the surgical care of cleft lip-cleft palate patients [2], while our survey results showed that 60.6% of the residents felt an improvement of the surgical skills. Besides the educational aspect, taking part of volunteering work surely enhance humanitarian values such as patriotism, solidarity, altruism and coming in rescue to people in need, it also develops sense of responsibility and also management of material resources [5, 7].

Among the surveyed participants, 92% wished to come back for another edition, because it was a free of charge and close at hand medical and surgical instruction. Unlike the other international missions, the resident doesn´t need to leave his hometown and family to purchase a humanitarian experience, a negative comment encountered in the Durning *et al*. work [5].

## Conclusion

In conclusion, the maxillofacial surgery department of Marrakesh, housing SOS FACE association, is individualized by the coupling of humanitarian action to the acquisition of technical skills and thus proves the educational value of these actions in the continuous development of the resident. These campaigns not only ensure surgical learning but also the development of more noble qualities: being human and humanitarian. We suggest that humanitarian work should be incorporated in residency programs.

### What is known about this topic

- Surgical training is at the heart of acquiring surgical skills;
- Residents volunteering in humanitarian missions.

### What this study adds

- The instructional value of humanitarian work in developing surgical skills;
- Paucity of evidences of this role in the literature;
- Sharing of our 10 years experience of structured humanitarian work coupled with targeted surgical training.

## Annex

**Annex 1**: example of survey given to participant in order to assess the educational outcome following a humanitarian mission
